# 0993. Heme oxygenase - 1 attenuates acute pulmonary inflammation by decreasing the release of segmented neutrophils from the bone marrow

**DOI:** 10.1186/2197-425X-2-S1-P78

**Published:** 2014-09-26

**Authors:** F Konrad, S Braun, I Vollmer, K-C Ngamsri, J Reutershan

**Affiliations:** University of Tübingen, Tübingen, Germany

## Introduction

The stress response enzyme heme oxygenase 1 (HO-1) is expressed ubiquitous in the body tissue and mediates, besides its major biological function of heme degradation, a variety of anti-inflammatory effects. Although these effects have been described in various models, underlying mechanisms remain elusive. Few studies revealed an influence of HO-1 on the bone marrow [1].

## Objectives

We investigated the particular role of the bone marrow in terms of HO-1-dependent pulmonary inflammation.

## Methods

Wildtype mice inhaled LPS for 30 minutes. After 24h, PMNs were detected in lung compartments (intravascular - interstitial - alveolar) using a flow cytometry-based technique. Bone marrow and blood were analyzed by flow cytometry after four hours. HO-1 was pharmacologically induced by Cobalt(III)Protoporphyrin-IX-Chloride (CoPP) and inhibited by TinProtophorphyrin-IX (SnPP). Chemokines were measured in the BAL and bone marrow by ELISA.

## Results

Immunohistochemistry revealed less lung destruction after HO-1 stimulation by CoPP, and *in vivo* migration assay showed reduced PMN migration into the BAL. Differentiation of PMNs in the BAL revealed a decrease of segmented PMNs after HO-1 activation. In this group, segmented PMNs were also decreased intravascularly and concordantly, mature and immature PMN populations were higher in the bone marrow. Inhibition of HO-1 by SnPP aggravated parameters of tissue inflammation, led to destruction and increased PMN migration into the BAL. Differential BAL counts in this group revealed an increase of segmented and banded PMNs with enhanced PMN release from the bone marrow. The chemokine SDF-1, which mediates homing of leukocytes into the bone marrow, was increased by CoPP and reduced by inhibition of HO-1. When SDF-1 was blocked by the specific antagonist AMD3100, HO-1 activation was no longer effective in curbing PMN trafficking to the inflamed lungs. Additionally, we compared the inhibition of HO-1 by SnPP alone and in combination with CoPP. Enzyme activity was more reduced after CoPP/SnPP treatment, resulting in deteriorated lung injury with higher PMN counts in the lung interstitium, and pronounced tissue destruction.

## Conclusions

We show evidence that the anti-inflammatory effects of HO-1 are largely mediated by inhibiting the release of segmented PMNs from the bone marrow rather than direct effects within the lung.
